# Prediction of blood pressure changes during surgical incision using the minimum evoked current of vascular stiffness value under sevoflurane anesthesia

**DOI:** 10.1038/s41598-023-46942-y

**Published:** 2023-11-22

**Authors:** Daiki Shorin, Satoshi Kamiya, Ryuji Nakamura, Ayaka Ishibashi, Noboru Saeki, Toshio Tsuji, Yasuo M. Tsutsumi

**Affiliations:** 1 Department of Anesthesiology and Critical Care, Hiroshima University Hospital, 1-2-3 Kasumi, Minami, Hiroshima, 734-8551 Japan; 2 Graduate School of Advanced Science and Engineering, Hiroshima University, Hiroshima, Japan

**Keywords:** Predictive markers, Biomedical engineering

## Abstract

Necessary and sufficient opioids should be administered for safe and stable anesthesia. However, opioid sensitivity varies among individuals. We previously reported that sympathetic responses to nociceptive stimuli under propofol anesthesia could be predicted by measuring the minimum evoked current of the vascular stiffness value (MEC_K_). However, this result has only been proven under propofol anesthesia. We propose that MEC_K_ could be used under anesthesia with a volatile anesthetic. Thirty patients undergoing laparotomy with sevoflurane anesthesia received 0.7 minimum alveolar concentration (MAC) sevoflurane and intravenous remifentanil at a constant concentration of 2 ng/mL, followed by tetanic stimulation, to measure MEC_K_. After tetanic stimulation, the same anesthetic conditions were maintained, and the rate of change in systolic blood pressure (ROC_BP_) during the skin incision was measured. The correlation coefficient between the MEC_K_ and ROC_BP_ during skin incision under sevoflurane anesthesia was R =  − 0.735 (*P* < 0.01), similar to that in a previous study with propofol (R =  − 0.723). Thus, a high correlation was observed. The slope of the linear regression equation was − 0.27, similar to that obtained in the study on propofol (− 0.28). These results suggest that, as with propofol anesthesia, MEC_K_ can be used as a predictive index for ROC_BP_ under 0.7 MAC sevoflurane anesthesia.

Clinical trial registration: Registry, University hospital Medical Information Network; registration number, UMIN000047425; principal investigator’s name, Noboru Saeki; date of registration, April 8, 2022.

## Introduction

Several pharmaceuticals are administered during anesthesia to induce unconsciousness (hypnosis and amnesia), analgesia, and immobility, including the inhibition of autonomic reflexes to noxious stimuli^1^. Various monitoring apparatuses have been introduced to establish adequate levels for each of these factors. Electrical stimulation, such as a train-of-four or post-tetanic counts, has been used to monitor immobilization^2^. Various electroencephalogram monitors such as the bispectral index (BIS), entropy, and SedLine are utilized in clinical practice despite various theories regarding the differences in measurement values depending on the type of anesthetic and the prediction accuracy for intraoperative awakening^3^. Some indicators for monitoring anesthesia have been proposed and are commercially available, such as the analgesia nociception index (ANI), nociception level (NOL), and surgical pleth index (SPI); however, whether their clinical significance is widely accepted is unclear^4–7^. During surgery, anesthesiologists estimate nociceptive stimuli based on surgical procedures, and they rely on changes in vital signs, such as BP, pulse, and electroencephalogram findings, to appropriately adjust the dose of analgesic medications^8^. Opioids are widely used as analgesics during the perioperative period because, in addition to their analgesic properties, they can suppress sympathetic nerve activity. However, overdoses of opioids can result in adverse events such as hypotension and bradycardia, which lead to hemodynamic disruption^9^. Furthermore, excessive intraoperative opioid use may trigger postoperative nausea and vomiting, and increase the risk of opioid-induced hyperalgesia developing, which may require that the subsequent dose for adequate analgesia be increased^10^. In contrast, if underdosage occurs, nociceptive stimuli can overstimulate the sympathetic nervous system, causing hypertension and tachycardia; it may even result in unwanted intraoperative wakefulness^11^. To avoid the adverse effects of opioids, a necessary and sufficient minimum dose should be administered; however, individual differences in opioid sensitivity make it challenging to use the same analgesic protocol for every patient. Various genetic polymorphisms have been cited as the cause of differences in individual opioid sensitivity. Using genetic testing to predict an individual’s opioid sensitivity has not yet been applied in clinical practice^12^. Additionally, patients who use opioids can develop opioid tolerance, resulting in changes in opioid sensitivity^10^. Therefore, to estimate an individual’s necessary and sufficient minimum dose, an opioid sensitivity index that can be measured quickly and easily in the operating room is needed.

Commercially available devices, such as the ANI, NOL, and SPI, are used in clinical practice to monitor analgesia^4–7^. These indices are useful for titrating opioid dosages by quantifying the body’s response to nociceptive stimuli^5,13^. The monitoring devices associated with these indices combine various autonomic nerve monitors, such as pulse photoplethysmography (PPG), skin conductance, and heart rate variability, and process them to quantify an individual’s sympathetic response to noxious stimuli^4–6^. The measured values of sympathetic responses vary greatly among individuals; however, comparing sympathetic responses based solely on changes in measured values is difficult^7^. These monitoring devices are primarily informed by changes in the patient’s own measurements, representing an increase or decrease in noxious stimuli within individuals, rather than an individual’s sensitivity to noxious stimuli. Therefore, when using these devices, predicting an individual’s opioid requirements—which is based on inter-individual opioid sensitivity differences—is challenging.

We have previously reported that vascular stiffness (K) values correlate well with noxious stimulus intensity^14–16^. However, similar to conventional indices that can be used to monitor analgesia, the K value varies among individuals and, as such, does not indicate opioid sensitivity for inter-individual comparisons^15^. Therefore, we proposed a minimum stimulus intensity value that evoked a response on K (MEC_K_), at which the K value changes in response to the minimal current and acts as an index of opioid sensitivity. Previously, MEC_K_ was strongly correlated with the rate of change in systolic blood pressure (ROC_BP_) during skin incision procedures under propofol anesthesia^17^. Additionally, the variation in measurements between individuals was small. In other words, MEC_K_ can be considered an individualized numerical value that predicts the strength of the sympathetic nerve response to noxious stimuli under constant opioid administration. This enabled us to infer that MEC_K_, which can be easily measured in the operating room, can be used as an indicator of an individual’s opioid sensitivity.

A previous study used propofol as the sedative. Volatile anesthetics such as sevoflurane are also widely used as sedatives during anesthesia. Sevoflurane is known to have an inhibitory effect on sympathetic nerve responses^18^, and further studies are needed to determine whether the MEC_K_ can be used as a predictive indicator of ROC_BP_ under sevoflurane anesthesia. The present study aimed to determine this.

## Methods

### Patients

The MEC_K_ and ROC_BP_ were measured in 30 adult patients who underwent laparotomy at Hiroshima University Hospital between April 2022 and February 2023. Included patients underwent general anesthesia with sevoflurane and required invasive arterial pressure measurement. Written informed consent was obtained from all patients prior to their participation. Patients with an irregular RR interval on electrocardiogram; those who were unable to undergo invasive arterial pressure measurements in the radial artery; those with significant hemodynamic or neurological impairments involving the upper extremities; those with severe stenotic or occlusive lesions in the coronary arteries or cerebral vessels; and those with contraindications to sevoflurane, remifentanil, or rocuronium were excluded.

This study adhered to the tenets of the Declaration of Helsinki and the STROBE statement. The study protocol was approved by the ethics committee of Hiroshima University (approval number, E-2180) and the study was registered as a clinical trial (registry, University hospital Medical Information Network; registration number, UMIN000047425; principal investigator’s name, Noboru Saeki; and date of registration, April 8, 2022).

### Measurement protocol

To measure the MEC_K_ and ROC_BP_ under the same conditions as in our previous study^17^, the following procedure was used in the present study: prior to inducing anesthesia, an electrocardiogram sensors for monitoring were placed on each patient’s chest, an oxygen saturation (SpO_2_) monitor (TL-271T; Nihon Kohden, Tokyo, Japan) on the left middle finger, a sensor of electroencephalogram monitor (GE Entropy Module; GE Healthcare UK Ltd., Buckinghamshire, UK) on the forehead, and a neuromuscular blockade monitoring device (E-NMT ; GE Healthcare UK Ltd., Buckinghamshire, UK) on the ulnar side of the forearm of the right hand. A dose of remifentanil was administered to achieve a predicted effect site concentration of 2 ng/mL based on Minto’s pharmacokinetic/pharmacodynamic model^19^, after which anesthesia was induced with 5% sevoflurane. In each patient, after confirming loss of consciousness, 50 mg of rocuronium was administered, a 22 G needle was placed in the left radial artery to monitor arterial pressure, and endotracheal intubation was initiated. Loss of consciousness was defined by loss of eyelash reflex. Thereafter, sevoflurane was administered again to achieve an expiratory concentration of 0.7 minimum alveolar concentration (MAC) after correcting for age^20^.

After sympathetic excitation caused by intubation-related stimulation subsided and the expiratory concentration of sevoflurane stabilized, continuous measurement of vascular stiffness (K value, explained in the next section) was initiated. Electrocardiogram, arterial blood pressure, and PPG data were output to a personal computer from a bedside patient monitor (BSS-9800; Nihon Kohden, Tokyo, Japan). These data were used to calculate the K values in real time. Tetanic stimuli were delivered at 50 Hz for five seconds through a two-pole body surface electrode on the ulnar side of the right hand using an Innervator 252 (Fisher & Paykel Healthcare, Auckland, New Zealand). The initial stimulation intensity was 10 mA and the intensity was increased in 10 mA increments to a maximum of 80 mA. A sufficient interval was provided between each stimulation. After confirming that the K value had returned to the pre-stimulus state, subsequent stimulation was performed. The same expiratory concentration of sevoflurane (0.7 MAC) and predicted effect-site concentration of remifentanil (2 ng/mL) were maintained until skin incision. The ROC_BP_ was measured.

### Calculation of K

The method for calculating the K values was previously reported by Nakamura et al.^14^ as follows. The vascular wall is assumed to be a mechanical impedance model consisting of a spring and damper. The respective coefficients are vascular stiffness (K) and viscosity (B). The pressure applied to the vessel wall is defined as the observed arterial pressure, the motion of the vessel wall as the amplitude of the PPG, t_0_ is the starting time of the change, and the arterial blood pressure and PPG amplitudes at time (t) were Pb(t) and Pl(t), respectively. Ṗl(t) is the first derivative of the PPG amplitude. The following relationship equation was established for K and B:

1 $$\begin{aligned} {\text{dPb}}\left( {\text{t}} \right) = & {\text{KdPl}}\left( {\text{t}} \right) + {\text{Bd}}\mathop {\text{P}}\limits^{.} {\text{l}}\left( {\text{t}} \right) \\ {\text{dPb}}\left( {\text{t}} \right) = & {\text{Pb}}\left( {\text{t}} \right) - {\text{Pb}}\left( {{\text{t}}_{0} } \right),\;\;{\text{dPl}}\left( {\text{t}} \right) = {\text{ Pl}}\left( {\text{t}} \right) - {\text{Pl}}\left( {{\text{t}}_{0} } \right) \\ {\text{d}}\mathop {\text{P}}\limits^{.} {\text{l}}\left( {\text{t}} \right) = & \mathop {\text{P}}\limits^{.} {\text{l}}\left( {\text{t}} \right) - \mathop {\text{P}}\limits^{.} {\text{l}}\left( {{\text{t}}_{0} } \right) \\ \end{aligned}$$

K and B were determined to be one value per heartbeat by performing a least-squares fit to Eq. (1). If the coefficient of determination was < 0.95, or if K and B were negative, the data were excluded from the analysis.

### Data processing

**Figure 1:**
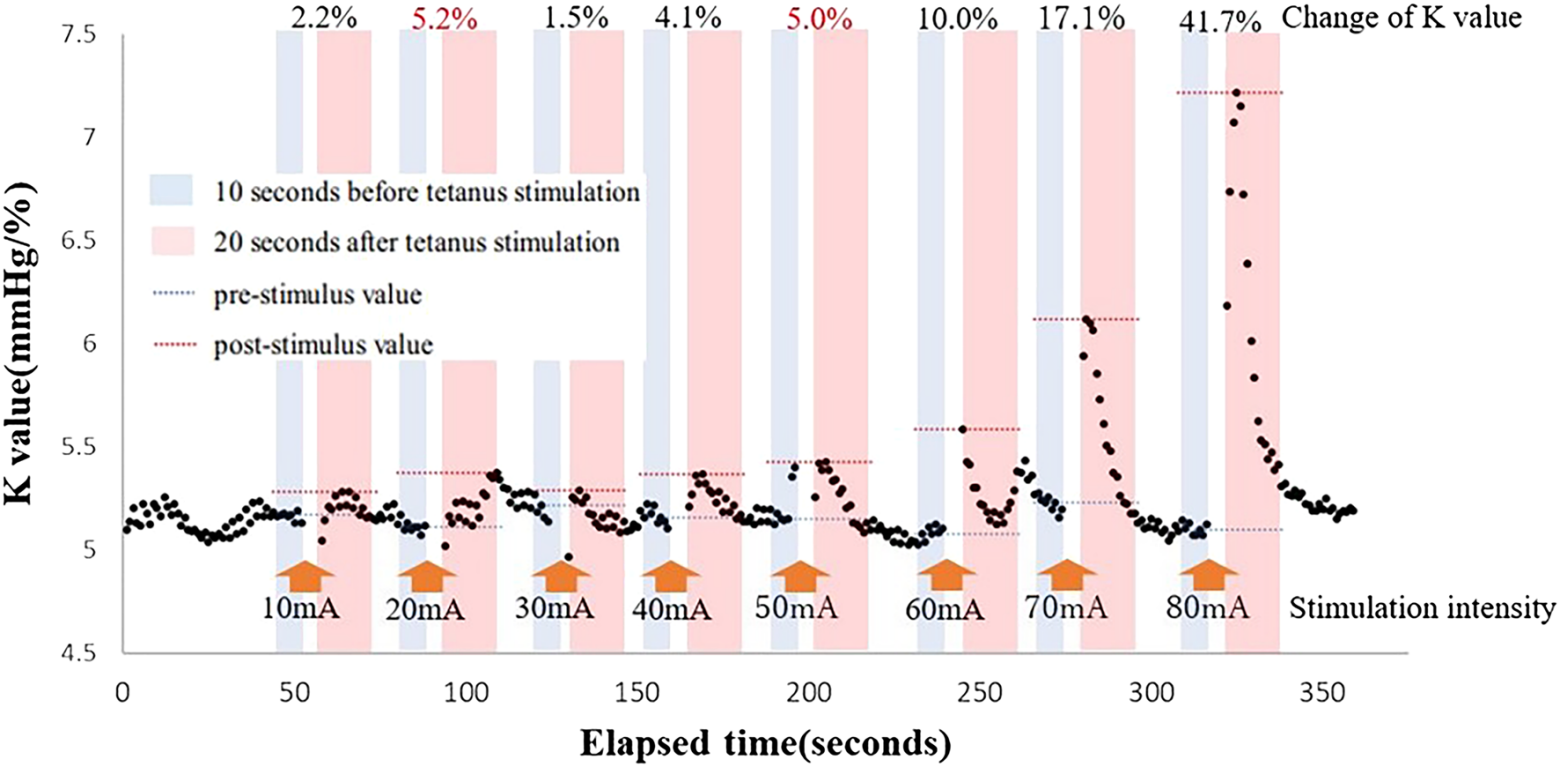
Variations in K value during MEC_K_ measurements. An example of the variations in the K value during tetanic stimulation for the MEC_K_ measurements is shown. In this case, the K value increases by > 5% after tetanic stimulation using 20 mA, but the increase is < 5% after stimulation using 30 mA. Therefore, the increase in the K value after 20-mA tetanic stimulation is considered noise. The actual value of K is assumed to be 50 mA. MEC_K_, minimum evoked current of the vascular stiffness value.

The MEC_K_ was measured as follows: the pre-stimulus K value was the median K value 10 s prior to stimulation, and the post-stimulus K value was the maximum K value 20 s after tetanic stimulation. The MEC_K_ was defined as the minimum stimulus intensity value at which K value began to increase. To remove noise due to fluctuations in biosignals, set a 5% threshold for the increase in K value from the pre-stimulus value. Even when the K value increased by ≥ 5%, if the rate of increase in the K value was < 5% in the subsequent stimulation, the previous stimulus value was rejected as noise. The MEC_K_ was classified as outside the measurement range if < 5% change occurred after tetanic stimulation using 80 mA. Figure 1 shows an example of K-value variation during tetanic stimulation. In case represented in Fig. 1, the MEC_K_ value was 50 mA.

For the ROC_BP_, the median systolic blood pressure (sBP) of 10 heartbeats prior to skin incision with a scalpel for electrocautery was defined as the pre-skin incision sBP. The maximum sBP from the surgical skin incision was defined as the post-skin incision sBP. The ROC_BP_ was calculated by dividing the post-skin incision sBP value by the pre-skin incision sBP value.

A first-order regression line for the MEC_K_ and ROC_BP_ was created for cases in which the MEC_K_ and ROC_BP_ could be measured. The predicted value of ROC_BP_ was calculated from the first-order regression line obtained, and the Smirnov–Grubbs test was performed to detect the outlier of the difference between the predicted and measured values with a *P*-value of < 0.05. Next, we evaluated the first-order regression lines of MEC_K_–ROC_BP_ obtained in our previous study^17^, which involved propofol-induced anesthesia, and in the present study using a parallel line test. Finally, we performed a Bland–Altman plot analysis of the predicted ROC_BP_ obtained by fitting the MEC_K_ measurements obtained in the present study to the prediction equation for ROC_BP_ obtained in our previous study^17^ and the ROC_BP_ measured in the present study.

### Outcomes

The primary outcome was the correlation coefficient between the MEC_K_ and ROC_BP_ under sevoflurane anesthesia.

### Statistical analysis

Power tests were performed using G*Power 3.1.0^21^. Our previous study involving propofol-induced anesthesia resulted in a coefficient of determination of 0.52 for the MEC_K_ and ROC_BP_ (coefficient of correlation, 0.72)^17^. In the present study, the ROC_BP_ was expected to be lower because of sevoflurane’s capability of suppressing sympathetic responses. Therefore, assuming a coefficient of determination of 0.25 for the present study (and a correlation coefficient of 0.5), the power analysis showed that the required number of cases was 26, subject to an α error of 0.05 and a β error of 0.2. As data collection errors were expected, the number of cases was set to 30. Pearson’s correlation analysis was used to calculate the correlation coefficients between the MEC_K_ and ROC_BP_ values.

In addition, subgroup analyses were performed to analyze the influence of patient factors on the results. First, we excluded thin patients (body mass index, < 20 kg/m^2^) whose pharmacokinetics differed from the average body size. The remaining patients with factors related to atherosclerosis—such as elderly patients (70 years or older); patients with hypertension, diabetes, hyperlipidemia, coronary artery disease, or chronic renal failure; and severely obese patients (body mass index, > 30 kg/m^2^)—were included in the atherosclerosis risk group^22^, while the other patients were included in the healthy group for subgroup analysis. In the subgroup analysis, Pearson’s correlation analysis was used to determine whether a significant correlation existed between the MEC_K_ and ROC_BP_ in each group. Parallelism of the regression lines for both groups was then evaluated using the test of parallel lines.

Patient demographics was presented with mean ± standard deviation (SD). *P* < 0.05 was considered statistically significant.

## Results

### Patient demographics

Thirty patients (22 males and 8 females) were enrolled in the present study; their demographics are presented in Table 1. The patient age was 63.7 ± 13.9 years, and the body mass index was 23.3 ± 4.0 kg/m^2^. Of the 30 patients, 23 underwent upper abdominal surgery and seven underwent lower abdominal surgery. The end tidal sevoflurane concentration was 1.1 ± 0.1% and the mean State Entropy was 52.8 ± 10.8. The pre-incision sBP was 89.7 ± 15.6 mmHg, which increased to 105.3 ± 17.0 mmHg post-incision. The pre-incision mean blood pressure (mBP) was 66.6 ± 11.2 mmHg, which increased to 78.0 ± 12.8 mmHg post-incision. None of the patients met the exclusion criteria.

**Table 1:** Patient demographics.

Male/female	22/8
Age (year)	63.7 ± 13.9(35–85)
Height (cm)	163.6 ± 9.7(145.7–180.5)
Weight (kg)	62.5 ± 12.8(40–95)
BMI (kg/m^2^)	23.3 ± 4.0(13.6–36.1)
Upper/lower abdomen	23/7
End tidal sevoflurane concentration (%)	1.1 ± 0.1(1.0–1.3)
State entropy	52.8 ± 10.8(24.0–84.5)
sBP of pre-incision (mm Hg)	89.7 ± 15.6(61.0–130.5)
sBP of post-incision(mm Hg)	105.3 ± 17.0(66.3–141.1)
mBP of pre-incision (mm Hg)	66.6 ± 11.2(49.4–91.5)
mBP of post-incision (mm Hg)	78.0 ± 12.8(53.7–101.4)
Rate of sBP's change before and after incision (%)	17.8 ± 7.6(5.6–34.10)

Results are presented as mean ±  standard deviation (SD; minimum–maximum). sBP, systolic blood pressure. mBP, mean blood pressure.

### MEC_K_

**Figure 2:**
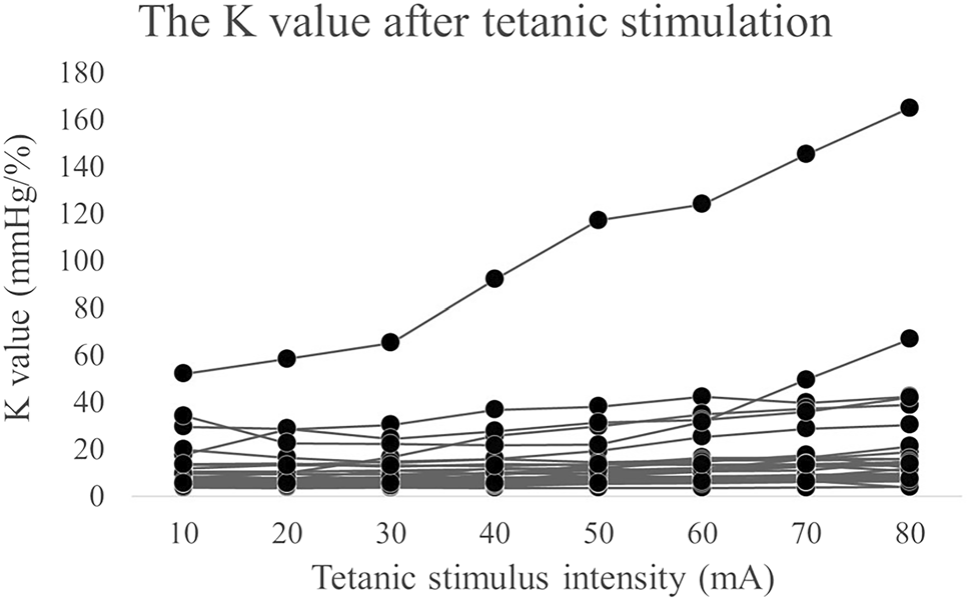
The K value after tetanic stimulation at each intensity. The maximum K value at 20 s after tetanic stimulation in increments of 10 mA in all cases are shown by the black dots. As the threshold value increases, so does the K value. One outlier has a significantly high K value at all thresholds.

The changes in K values based on the MEC_K_ measurements from each patient are shown in Fig. 2. In most cases, the K value increased as the stimulus intensity increased. In one case, the K values were abnormally high from the beginning of the MEC_K_ measurement and increased to over 160 mm Hg/% after tetanic stimulation using 80 mA.

**Figure 3:**
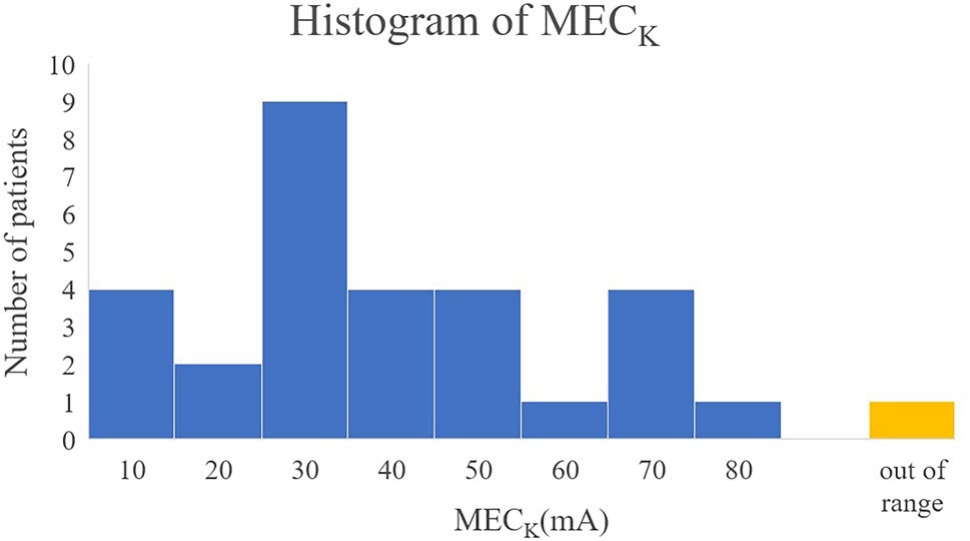
Histogram of the MEC_K_. Distribution of each type of MEC_K_ is shown. The most frequent threshold current is 30 mA and more than half the patients have a threshold current of between 20 and 40 mA. MEC_K_, minimum evoked current of the vascular stiffness value.

The MEC_K_ was measured in 29 of the 30 patients. In one case, the K values never increased by > 5%, even with an 80-mA stimulation, and were therefore considered out of range. The distribution of the MEC_K_ values is shown in Fig. 3, indicating that 30 mA was the most common threshold. However, more than half the patients had a threshold between 20 and 40 mA.

### The correlation coefficient between the MEC_K_ and ROC_BP_

**Figure 4:**
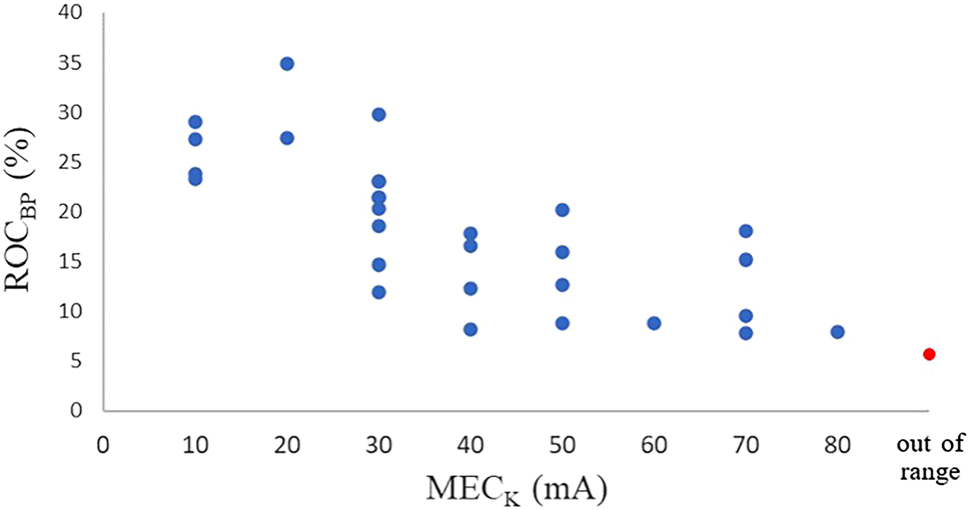
Relationship between the MEC_K_ and ROC_BP_. Scatter plots of the MEC_K_ and ROC_BP_ in the present study is shown. In this study, one patient was out of range, which means that the MEC_K_ exceeded 80 mA, as shown by the red dot. A downward rightward trend is observed. MEC_K_, minimum evoked current of the vascular stiffness value; ROC_BP_, rate of change in systolic blood pressure.

When examining the relationship between the MEC_K_ and ROC_BP_, a downward trend was observed as the MEC_K_ increased (Fig. 4). The correlation coefficient between the MEC_K_ and ROC_BP_ was − 0.735 (*P* < 0.01) for 29 patients, excluding the patient for whom the MEC_K_ was outside the measurement range. The regression equation obtained from the first-order regression line of the MEC_K_ and ROC_BP_ under sevoflurane anesthesia was as follows:

2 $${\text{ROC}}_{{{\text{BP}}}} \left( \% \right) = - 0.{27} \times {\text{MEC}}_{{\text{K}}} \left( {{\text{mA}}} \right) + {28}.{81}$$

Finally, Smirnov–Grubbs tests were performed using Eq. (2) and the measured values, none of which were excluded as outliers.

### The influence of patient factors

Subgroup analysis was performed on nine patients in the healthy group and 17 patients in the atherosclerosis risk group. The relationship between the MEC_K_ and ROC_BP_ showed a downward-rightward trend in both groups, similar to that observed in all cases. The correlation coefficients between the MEC_K_ and ROC_BP_ were − 0.893(*P* < 0.01) and − 0.631(*P* < 0.01), respectively, indicating significant correlation in both groups. When comparing the two regression lines, the parallelism in the two lines was not rejected (*P* = 0.058) and no significant difference in the intercept was observed (*P* = 0.628).

## Discussion

We found a strong relationship between the MEC_K_ and ROC_BP_ under sevoflurane anesthesia. The absolute value of the correlation coefficient between the MEC_K_ and ROC_BP_ was as high as 0.735 (*P* < 0.01), and the Smirnov–Grubbs test showed no outliers, indicating that the MEC_K_ was a good predictive indicator of the ROC_BP_. The MEC_K_ may be a good indicator for assessing individual opioid sensitivity, even under sevoflurane anesthesia.

Yanabe et al.^15^ showed substantial individual differences in absolute K values, whereas our previous study^17^ showed that, under propofol anesthesia, MEC_K_ alone could predict BP variability with high accuracy, indicating that MEC_K_ is an index with few individual differences. Both sevoflurane and propofol are known to have the effect of relaxing vascular smooth muscle, but this vascular smooth muscle relaxing effect is stronger due to sympathetic nerve inhibitory effect than the direct relaxation effect^23,24^. This suggests that the sympathetic nerve inhibitory effect of sevoflurane and propofol may influence the measurement of MEC_K_. However, the minimum alveolar concentration blocking adrenergic response (MAC_BAR_) of sevoflurane is as high as 8.0%^25^ and sevoflurane alone is known to have a low inhibitory effect on sympathetic nerve response. Similarly, the inhibitory effect of propofol alone on sympathetic nerve response is also known to be poor^26^. However, opioids inhibit nociceptive stimulation and decrease the MAC_BAR_. The MAC_BAR_ of sevoflurane with 2 ng/mL fentanyl is known to be approximately 1.1%^27^. Remifentanil is said to have approximately 1.2 times the medicinal effect of fentanyl^28^, and the MAC_BAR_ of sevoflurane in the setting of the current study may be similar to 1.1%. Therefore, under the conditions of this study, the sympathetic response to nociceptive stimulation by sevoflurane would have been inhibited in approximately half of the cases. However, unlike the absolute value of K, the MEC_K_ measures the absence of a sympathetic response rather than the strength of a sympathetic response. The fact that the MEC_K_ showed a high correlation with the ROC_BP_ suggesting that the mild inhibitory effect of propofol and sevoflurane on sympathetic responses had little impact on the MEC_K_.

**Figure 5:**
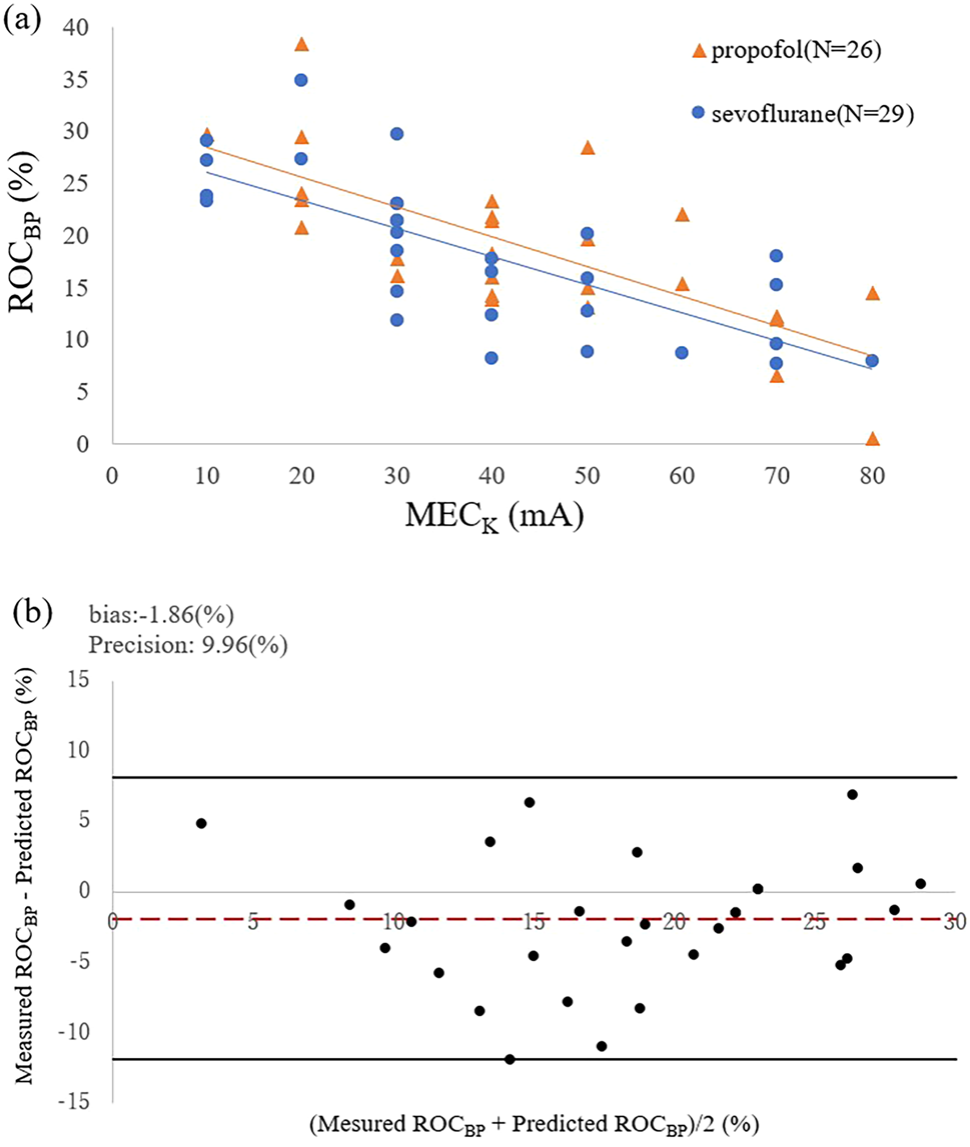
Comparison with our previous study^17^ in which propofol anesthesia was used. A scatter plot of the MEC_K_ and ROC_BP_ and the linear approximation of both the present (sevoflurane anesthesia) and previous (propofol anesthesia) studies are shown in (**a**). A downward rightward trend is observed, with similar slopes for the approximation lines. The intercept of sevoflurane is lower than that of propofol; however, the difference is not significant. The errors between the predicted and measured ROC_BP_ values are shown in (**b**). The measured ROC_BP_ values are those obtained in the present study. The predicted ROC_BP_ values were calculated from the MEC_K_ values obtained in this study and the prediction equation obtained in a previous study. The fixed and proportional errors are minimal. MEC_K_, minimum evoked current of the vascular stiffness value; ROC_BP_, rate of change in systolic blood pressure.

The regression equation obtained from the first-order regression line of the MEC_K_ and ROC_BP_ under sevoflurane anesthesia was close to that obtained under propofol anesthesia in our previous study; therefore, we performed a test of parallel lines of the first-order linear regression equation in both studies (Fig. 5a). Parallelism in the first-order linear regression equation was not rejected (*P* = 0.851) and no significant difference was found in the intercept (*P* = 0.193). Similarity between the linear regression lines under 0.7 MAC sevoflurane and propofol anesthesia was demonstrated, suggesting that these equations are interchangeable. Therefore, we fit the data from the present study to the prediction equation for the ROC_BP_ in our previous study and performed a Bland–Altman plot analysis (Fig. 5b). At − 1.86%, fixed bias was minimal, and the precision range was almost the same (9.96% in the present study vs. 10.17% in the previous study). These findings suggest that the prediction equation for the ROC_BP_ under propofol anesthesia can be successfully used for ROC_BP_ under 0.7 MAC sevoflurane anesthesia. As the prediction equation for the ROC_BP_ under propofol anesthesia showed almost the same accuracy in the prediction of the ROC_BP_ using different data, the validity of the prediction equation itself was demonstrated. We expected that sevoflurane would also exhibit analgesic properties, which may have suppressed changes in both the MEC_K_ and ROC_BP_. However, we found no significant differences in either the MEC_K_ or ROC_BP_ compared with previous study using propofol^17^. This may be due to the relatively low concentration of sevoflurane used in the present study (0.7 MAC), in which sevoflurane was primarily used for loss of consciousness. The minimum alveolar concentration-awake of sevoflurane is 0.6%^29^, a value which does not change significantly with increasing concentrations of fentanyl^30^. Therefore, in the present study, we used the lowest dose of sevoflurane necessary to guarantee loss of consciousness, and remifentanil was primarily responsible for the analgesic effect. The contribution of sevoflurane to the inhibition of the sympathetic nerve response was assumed to be low, resulting in a linear regression equation similar to that of propofol.

Aging and arteriosclerosis are known to affect vascular reactivity^31,32^. Therefore, we compared the healthy group with the atherosclerosis risk group. The results from this analysis suggested that the presence or absence of arteriosclerotic risk factors had little effect on the MEC_K_–ROC_BP_ relationship. This lack of effect is caused by the fact that, unlike the absolute value of K, the MEC_K_ measures the presence or absence of sympathetic response. Arteriosclerosis reduces vascular reactivity; however, the MEC_K_, which measures the onset of response, is a good predictor of the ROC_BP_, regardless of the presence or absence of arteriosclerotic factors.

The present study has a few limitations. First, the K values were measured using PPG amplitude and arterial pressure measurements, which can be challenging to use in clinical practice because of their invasive nature. Second, although MEC_K_ has been suggested as a good indicator of an individual’s opioid sensitivity, whether changing the opioid dosage depending on an individual’s MEC_K_ reduces circulatory responses to noxious stimuli remains unclear. To clarify this, we intend to conduct a study in which the opioid dosage is varied depending on an individual’s MEC_K_. Additionally, the size of the K values from the PPG and the observed arterial pressure require dedicated recording and analysis equipment. Third, remifentanil in known to have a direct effect on the arterial muscles^33^. However, the impact of this effect on the relationship between the MEC_K_ and ROC_BP_ of the present study is unclear. Further studies comparing remifentanil with other analgesics are needed to clarify this. Finally, the MEC_K_ was measured every 10 mA. If the MEC_K_ can be measured at smaller intervals, the prediction accuracy of the ROC_BP_ may improve.

In conclusion, the MEC_K_ can be used as a predictive index for the ROC_BP_ under 0.7 MAC sevoflurane anesthesia. Additionally, the prediction equations for the ROC_BP_ obtained under propofol anesthesia showed similar predictive performance for the ROC_BP_ obtained under 0.7 MAC sevoflurane anesthesia.

## Data Availability

The datasets used and/or analyzed in the present study are available from the corresponding author upon reasonable request.
